# Latest Trends in Acoustic Sensing

**DOI:** 10.3390/s140405781

**Published:** 2014-03-25

**Authors:** Cinzia Caliendo

**Affiliations:** Istituto di Acustica e Sensoristica “Orso Mario Corbino”, IDASC-CNR, Via del Fosso del Cavaliere 100, 00133 Roma, Italy; E-Mail: cinzia.caliendo@idasc.cnr.it; Tel.: +39-06-4548-8741; Fax: +39-06-4548-8061

Acoustics-based methods offer a powerful tool for sensing applications. Acoustic sensors can be applied in many fields ranging from materials characterization, structural health monitoring, acoustic imaging, defect characterization, *etc.*, to name just a few. A proper selection of the acoustic wave frequency over a wide spectrum that extends from infrasound (<20 Hz) up to ultrasound (in the GHz–band), together with a number of different propagating modes, including bulk longitudinal and shear waves, surface waves, plate modes, etc., allow acoustic tools to be successfully applied to the characterization of gaseous, solid and liquid environments. The purpose of this special issue is to provide an overview of the research trends in acoustic wave sensing through some cases that are representative of specific applications in different sensing fields.

## Summary of the Special Issue

A methodology to quantify bolt tension is described in Reference [1]: the reflection of surface acoustic waves (SAWs) generated by a phased array is used to estimate bolt tension without any contact with the bolt. Real contact areas formed in-between the bolt head and the clamped elements are imaged using a synthetic phase array. SAWs are generated and sensed by off-the shelf bulk piezoelectric transducers connected to a custom-designed ultrasonic wedge. The experimentally obtained results show that the proposed method is capable of clearly distinguishing properly bolted joints from loosened joints, and it is also capable of quantifying how loose the bolt actually is. The analysis of the signal-to-noise performed for the entire bolt tension range shows that it doesn't affect the image reconstruction. The proposed method has a potential to be used as a component in a non-destructive structural health monitoring system for local monitoring of civil infrastructures.

The propagation of the S_0_ Lamb mode along β-SiC/c-AlN composite plates is theoretically studied in Reference [2] for application to the design of temperature-compensated, enhanced-coupling, GHz-range gravimetric sensors. The theoretically predicted SiC/AlN-based sensor performances are compared to those of surface acoustic waves and Lamb S_0_ mode mass sensors implemented on bulk conventional piezoelectric materials and on thin suspended membranes. The AlN/SiC-based mass sensors are proven to achieve remarkable performances (high sensitivity, low resolution values, thermostability and enhanced coupling efficiency) that are important prerequisites for the design of future devices, based on resonator principles, to be used in the context of chemical, biological and physical quantities detection.

In Reference [3] a statistical propagation model is developed in which the transmission loss in time-varying and location-sensitive acoustic environment, such as an underwater channel, is treated as a random variable. The random variations are analyzed in the large-scale transmission loss that are mainly governed by environmental factors, such as surface activity (waves) for a particular network scenario. For first the deterministic prediction model based on the Bellhop ray tracing tool is applied resulting in accurate results for a specific geometry of the system, but without reflecting the variations that occur as the geometry changes slightly, due to either surface motion or transmitter/receiver motion. The authors employ an existing deterministic prediction model, such as the ray tracer, to generate an ensemble of channel responses corresponding to varying propagation conditions in a given network scenario. The so-obtained values were used in a statistical analysis to obtain the probability density function of the large-scale transmission loss to be employed for network design and analysis.

In Reference [4] an acoustic waveguide sensor based on multiple mode conversion of surface acoustic waves at the solid–liquid interface is studied, suitable for the concentration measurement of binary and ternary mixtures, liquid level sensing, investigation of spatial inhomogeneities or bubble detection. A single phase transducer at 1 MHz excites the propagation of the antisymmetrical zero order Lamb wave mode on a thin glass plate that is in contact with a liquid and forms an acoustic waveguide with a second glass plate opposite the first plate. The acoustic field of the zigzag sound propagation pathway through the liquid gap between the two plates is visualized by Schlieren imaging for continuous and burst operation.

A multiparametric biosensor system for the investigation of living cells is presented in Reference [5]. The system includes two different sensing techniques on a single device; resonant frequency measurements based on a quartz crystal microbalance (QCM) resonator and electric impedance sensing. Bovine aortic endothelial live cells (BAECs) have been cultured on the hybrid biosensor and simultaneous gravimetric and impedimetric measurements have been performed over a period of time on the same cell culture.

In Reference [6] an indoor positioning system based on time-of-flight (TOF) of ultrasonic signal is described that estimates the distance between a receiver node and a transmitter node. The system, named TELIAMADE, consists of a set of wireless nodes equipped with a radio module for communication and a module for the transmission and reception of ultrasound. The access to the ultrasonic channel is managed by applying a synchronization algorithm based on a time-division multiplexing (TDMA) scheme. Experimental results show a root mean square error less than 2 mm and a standard deviation less than 0.3 mm for pseudorange measurements in the range of distances between 2 and 6 m. A sub-centimeter location accuracy is achieved with an average rmse of 9.6 mm.

In Reference [7] a blind coherent two-dimensional direction of arrival (2D-DOA) estimation algorithm for arbitrarily spaced acoustic vector-sensor arrays subject to unknown locations is presented. The algorithm combines the acoustic vector-sensor array parameter estimation problem with the parallel profiles with linear dependencies (PARALIND) model, originally applied to biology and chemistry. The proposed algorithm achieves automatically paired azimuth and elevation angles for coherent and incoherent angle estimation of acoustic vector-sensor arrays, as well as the paired correlated matrix of the sources.

In Reference [8] Spectral Analysis of Surface Waves (SASW) is used to detect subsurface properties for the inspection of small-scale structures, such as concrete slabs, pavements and subsurface profile of roadways, with the additional effort to replace the traditional surface-mounted transducers with non-contact acoustic transducers. A Mobile Acoustic Subsurface Sensing system, MASS, has been developed consisting of a 3-wheeled cart outfitted with an electromagnetic impact source, distance register, non-contact acoustic sensors and data acquisition/ processing equipment. The MASS system is capable to collect measurements continuously at walking speed in an automatic way. The fast scan and real-time analysis advantages are based upon the non-contact acoustic sensing and fast air-coupled surface wave analysis program. This integration of hardware and software makes the MASS system an efficient mobile prototype for the field test.

In Reference [9] a Stratified Acoustic Model (SAM) is proposed for large scale underwater acoustic network simulation, based on frequency-independent geometrical ray tracing, accounting for each ray's phase shift during the propagation. It allows to predict the transmission loss with much lower computational complexity than the traditional physics-based models. The accuracy of the model is validated via comparisons with the experimental measurements in two different oceans. Satisfactory agreements with the measurements and with other computationally intensive classical physics-based models are demonstrated.

Reference [10] addresses the problem of three-dimensional speaker orientation estimation in a smart-room environment equipped with microphone arrays. A Bayesian approach is proposed to jointly track the location and orientation of an active speaker. Assuming that the sound produced by the speaker is originated from his mouth, the center of the head is deduced based on the estimated head orientation. Moreover, the elevation angle of the head of the speaker can be partly inferred from the fast vertical movements of the computed mouth location. In order to test the performance of the proposed algorithm, a new multimodal dataset has been recorded for this purpose, where the corresponding 3D orientation angles are acquired by an inertial measurement unit (IMU) provided by accelerometers, magnetometers and gyroscopes in the three-axes. The proposed joint algorithm outperforms a two-step approach in terms of localization and orientation angle precision assessing the superiority of the joint approach.
